# Cyclopœdia Diseases of Children; Keating; Vol. II

**Published:** 1889-10

**Authors:** 


CYCLOPOEDIA OF DISEASES OF CHILDREN, MEDICAL AND SURGICAL. Keating. Vol. II, Illustrated. Diseases of the Skin. Constitutional Diseases and Diseases of Nutrition; of the Respiratory, Circulatory, Haemato Poietic and Glandular Systems and of the Mouth, Tongue and Jaw. 1066 pages, cloth boards, Philadelphia, J. B. Lippincott & Co., 1889.

When it is remembered that each chapter in this masterly work was written by a specialist eminent in his line of study, and the vast scope set forth above is considered, some idea may be had of the value and importance of the work. Even were this one volume alone sent out, it would be a great help to the practitioner, for it contains an enormous amount of well digested matter set out in attractive style. Having written an elaborate description of the antecedent volume—on the footsteps of which this has followed so rapidly, we deem it unnecessary to do more than announce its advent, with an outline as above, of the treat prepared for its lucky subscribers. The price we believe is $5 per volume, in cloth. The full set will be a treasurer.

